# 3-D Modelling of Megaloolithid Clutches: Insights about Nest Construction and Dinosaur Behaviour

**DOI:** 10.1371/journal.pone.0010362

**Published:** 2010-05-05

**Authors:** Bernat Vila, Frankie D. Jackson, Josep Fortuny, Albert G. Sellés, Àngel Galobart

**Affiliations:** 1 Institut Català de Paleontologia, Campus de la Universitat Autònoma de Barcelona, Barcelona, Spain; 2 Department of Earth Sciences, Montana State University, Bozeman, Montana, United States of America; Raymond M. Alf Museum of Paleontology, United States of America

## Abstract

**Background:**

Megaloolithid eggs have long been associated with sauropod dinosaurs. Despite their extensive and worldwide fossil record, interpretations of egg size and shape, clutch morphology, and incubation strategy vary. The Pinyes locality in the Upper Cretaceous Tremp Formation in the southern Pyrenees, Catalonia provides new information for addressing these issues. Nine horizons containing *Megaloolithus siruguei* clutches are exposed near the village of Coll de Nargó. Tectonic deformation in the study area strongly influenced egg size and shape, which could potentially lead to misinterpretation of reproductive biology if 2D and 3D maps are not corrected for bed dip that results from tectonism.

**Methodology/Findings:**

Detailed taphonomic study and three-dimensional modelling of fossil eggs show that intact *M. siruguei* clutches contained 20–28 eggs, which is substantially larger than commonly reported from Europe and India. Linear and grouped eggs occur in three superimposed levels and form an asymmetric, elongate, bowl-shaped profile in lateral view. Computed tomography data support previous interpretations that the eggs hatched within the substrate. Megaloolithid clutch sizes reported from other European and Indian localities are typically less than 15 eggs; however, these clutches often include linear or grouped eggs that resemble those of the larger Pinyes clutches and may reflect preservation of incomplete clutches.

**Conclusions/Significance:**

We propose that 25 eggs represent a typical megaloolithid clutch size and smaller egg clusters that display linear or grouped egg arrangements reported at Pinyes and other localities may represent eroded remnants of larger clutches. The similarity of megaloolithid clutch morphology from localities worldwide strongly suggests common reproductive behaviour. The distinct clutch geometry at Pinyes and other localities likely resulted from the asymmetrical, inclined, and laterally compressed titanosaur pes unguals of the female, using the hind foot for scratch-digging during nest excavation.

## Introduction

The titanosaur clade has long been associated with eggs of the oofamily Megaloolithidae [1]–[6]. The most extensively documented megaloolithid localities occur in Upper Cretaceous rocks of southern France, northern Catalonia, India, and South America [7]. Although some authors (e.g., Grigorescu et al., [8], [9]) referred *Megaloolithus* eggs from Romania to a hadrosaur, this assignment remains unsubstantiated by a detailed description of the eggs and their association with osteological remains. Furthermore, the 1997 discovery of titanosaur embryos in *Megaloolithus patagonicus* eggs from the Auca Mahuevo locality in Argentina allowed the first definitive assignment of *Megaloolithus* eggs to titanosaur sauropod dinosaurs [10], [11].

This extensive and worldwide fossil record of megaloolithid eggs provides important data for assessing reproductive characteristics such as egg size and shape, clutch morphology, and incubation strategy. However, interpretations often differ. For example, descriptions of clutch size vary from 1 to over 40 eggs [7], [12] and reported egg shapes include spherical, sub-spherical, and elliptical [12]–[21]. Inferences of nesting strategies employed by dinosaurs also differ. Some authors suggest that the eggs were laid on or near the ground surface, possibly in vegetation mounds [15], [22], [23]. Most studies, however, conclude that megaloolithid eggs were buried in a substrate, typically described as an excavated pit (e.g. [6], [24]–[31]). Most water vapour conductance (G_H20_) studies of European megaloolithid eggs also support interpretations of egg burial (e.g. [32]–[36]). However, trace fossil nests [37] and G_H20_ calculated for the Auca Mahuevo titanosaur eggs in Argentina [36] suggest that these eggs were not incubated underground and the strategy employed remains unclear.

Controversial interpretations about titanosaur reproductive biology often arise from the lack of detailed taphonomic studies conducted at many fossil egg localities. With the exception of the six nesting structures preserved at Auca Mahuevo [37], nesting traces are unknown at any megaloolithid site. Typically, the eggs occur in fine-grained, homogeneous substrates that lack lithologic evidence of nest excavation. This hinders interpretation of nest structure and clutch geometry. Furthermore, most previous studies that use two-dimensional mapping of eggs fail to note and correct for the dip of the beds when reconstructing clutch morphology and egg arrangement (e.g., [1], [3], [6], [8], [15], [20], [25], [27], [28], [38], [39]). This is important because 2-D maps of excavations that cross-cut the bedding plane may fail to accurately portray egg shape and the biological pattern of egg distribution. Chiappe et al. [12] provided the first 3-D reconstruction of egg and clutch distribution; however, only three studies include 3-D reconstructions for an entire egg clutch [31], [40], [41]. Here, we use detailed 3-D modelling to document eggs and clutches at the Pinyes locality near the village of Coll de Nargó, in Lleida Province, Catalonia. The new 3-D modelling technique provides insights about egg and clutch morphology, site taphonomy, and the reproductive behaviour of dinosaurs producing megaloolithid eggs.

### Geological Setting

The evolution of the south-Pyrenean basin was controlled by the placement of the Pyrenean thrusts [42] from Maastrichtian to Oligocene time [43], [44]. Sediments of this basin are exposed today in the allocthonous western, central and eastern tectonic structures, which include the Tremp, Àger, Coll de Nargó and Vallcebre synclines. In latest Cretaceous time the south-Pyrenean basin was open to the sea in the western sector, and the continental sediments progressively graded into marine environments [45]-[48]. Two dinosaur-bearing formations record this regressive episode: the Arén Sandstone and the Tremp Formation. The Arén Sandstone interfingers with the lowermost deposits of the Tremp Formation and records latest Campanian-Maastrichtian coastal environments.

The Tremp Formation [49], historically referred to as the “Garumnian” facies of the southern Pyrenees (see review in [47]), is composed of continental deposits recording the Cretaceous-Tertiary transition. During the last century the Tremp Formation produced most of the fossil vertebrate remains in the basin (see references in [48], [50]). The formation includes three informal units ([47] and references therein): 1) a lower grey unit that includes coals, grayish calcareous mudstones, and limestones; 2) a lower red unit that consists of reddish mudstones intercalated with small to medium-sized sandstone bodies containing mottling, caliche nodules, and evidence of extensive bioturbation; and 3) an upper red unit, which includes the lacustrine “Vallcebre Limestone”, mudstones, sandstones, conglomerates, and other limestones deposited in various continental environments. The age of the two lower units varies from late Campanian to entirely Maastrichtian, whereas the third unit is considered as early Paleocene [48], [51], among others).

### The Pinyes Locality

**Figure 1:**
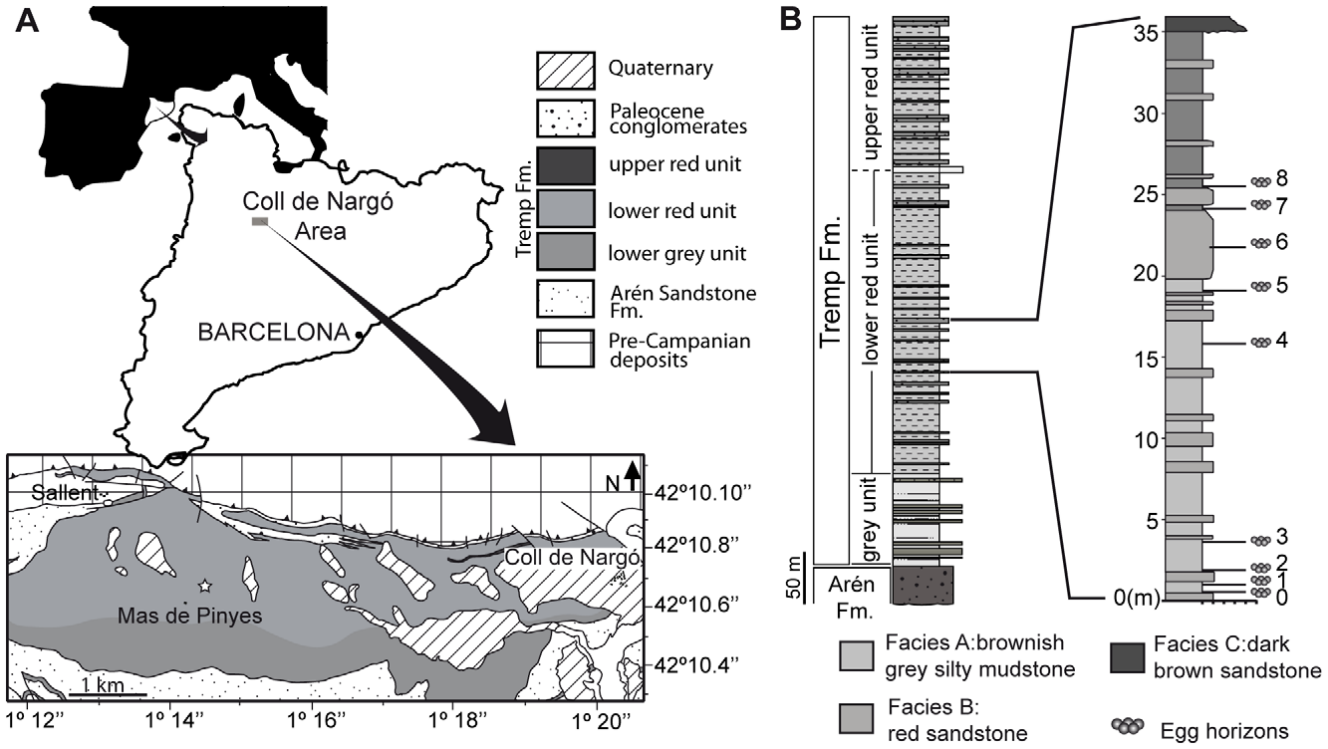
General setting of the Pinyes locality. (A)–Geographical and geological location of the locality within Catalonia and the Coll de Nargó nesting area. (B)–Stratigraphical section of the Pinyes locality highlighting the nine egg horizons (see details of sedimentary facies in [52], [53]).

The Upper Cretaceous outcrops of the Tremp Formation that are present at the Coll de Nargó syncline are limited by the Montsec thrust to the south and by the Bóixols thrust to the north (Fig. 1A). Stratigraphically, the Pinyes locality falls in the lower portion of the lower red unit of the Tremp Formation (Fig. 1B). Three lithofacies identified at the Pinyes locality include pedogenically modified massive, calcareous silty mudstones, very fine to fine-grained sand bodies, and medium to coarse-grained sandstone [52], [53]. The rocks comprising the local section are interpreted as sedimentary deposits of a fluvial environment that were located some distance from an active stream channel.

## Materials and Methods

### Field Data Acquisition

Table 1 summarizes the horizons, sites, and egg clusters at the Pinyes locality. With the exception of 17E04, which contains 2 clusters (designated as A and B), all other sites contain only one cluster, and therefore the assigned number identifies both the site and egg cluster. It should also be noted that three horizons (0, 2, 4) contain fossil eggs but remain largely unexplored.

**Table 1:** Pinyes eggs and clusters.

Egg horizon	Site	Eggs per cluster	Egg arrangement (plan view)	Number of egg levels
1	13E01	5	-	-
3	13E02	>3	-	-
5	17E02	2	-	-
	17E03	10	linear	-
	17E04	A = 8	grouped	2
		B = 20	linear+grouped	3
	17E05	18	randomly dispersed	-
	17E06	5	grouped	2
6	18E04	11	linear+grouped	2
7	18E01	9	Grouped	2
8	18E02	28	linear+grouped	3

Three horizons (0, 2, 4) exhibit fossil material but remain unexplored. Note: Horizons 5–8 are equivalent to horizons 1–4 in [52], [53].

A stratigraphic section was drawn for a 36 m-thick interval that contains the egg-bearing horizons (Fig. 1B; [52], [53]). Small hand tools and pneumatic jackhammers facilitated exposure of the eggs, which were then mapped using a metric grid and graph paper, with strike and dip direction noted on the map. A Series 5000 Trimble Total Station was used to collect data points (x, y, z coordinates) around each egg (total points >1,700) in order to document the precise location of eggs in 8 clusters; additional data points (∼200) from 6 eggs in cluster 18E02 were collected after further preparation of the specimen. These data points allowed high-resolution mapping of eggs in each cluster, using Rhinoceros® 4.0 3-D analysis software. Taphonomic data were collected from each site (Table 1), and specimens photographed using Olympus C-750 and PENTAX Z10 digital cameras. Where possible, data were recorded for both plan and cross sectional views of exposed eggs. The egg clusters were covered with aluminum foil and surrounded by cardboard panels prior to the application of a 2-part polyurethane foam that provided protection during transport. For further analyses, eggshell fragments were removed from eggs in seven clusters (e.g., 412 samples from five eggs in cluster 17E04, 548 samples from three eggs in cluster 17E05, 612 samples in cluster 18E01 and 51 samples in cluster 18E03).

### Laboratory Analyses

Eggshell fragments were washed in a solution of sodium hydroxide phosphate, submerged in an ultrasound bath, and then examined and photographed using a Leica® MZ16A stereomicroscope. Additional samples were freshly broken and half of each fragment coated with 10 nm of gold, mounted on aluminum stubs, and imaged at 15 kV with a J.R. Lee Instrument Personal SEM. The other half of each sample was prepared as a standard petrographic thin section, 30 µm thick, and studied using a Nikon LV100POL light microscope. Microstructural features were measured from digital images with Leica® Application Suites 2.8.1 software or Scion Image Analysis software.

Fourteen eggs in six clusters (13E01C, 13E01D, 13E01E; 13E02A, 13E02B, 13E02C; 17E04P; 18E01A, 18E01B, 18E01C, 18E01D, 18E01E, 18E01H; 18E02E) were scanned with a CT Siemens® Sensations-16, at 140 kV and 350 mAs obtaining an output of 512×512 pixels per slice for all the specimens. The pixel size and the inter-slice space were 0.529 mm and 0.3 mm, respectively, for 13E01C, 13E01D, 13E01E; 0.5 mm and 1 mm for 13E02A; 0.477 mm and 0.2 for 13E02B; 0.586 mm and 0.2 mm for 13E02C; 0.586 mm and 0.3 mm for 17E04P; 0.977 mm and 0.3 mm for 18E01A, 18E01B, 18E01C, 18E01D, 18E01E, 18E01H; and 0.391 mm and 0.3 mm for 18E02E.Mimics® software provided 3-D models and 2-D slices of each specimen. These CT scans provide additional information on the distribution of eggshell within the matrix that fills the eggs.

Eggs were modelled as scalene ellipsoids (mean semi-principal axes X = 10.5 cm, Y = 8 cm, Z = 5 cm.) by using the Trimble Total Station data set. These data were augmented by field measurements, taphonomic observations, and cross-sections views provided by CT scans. A three-dimensional model for each of the 8 egg clusters was then generated using Rhinoceros® 4.0 software. A horizontal surface was created for the bedding plane by joining all the measured topographic points, thereby showing the actual dip of the layer (see [31], [41] for methods). As a result, the 3-D model allows visualization of the egg positions from any perspective. Long axis direction of eggs (n = 63) were plotted by using Rose 2.1.0 software.

### Terminology

Egg horizon, a stratigraphic bed containing *in situ* egg remains (eggshells, eggs, or clutches); site, a specific location containing one or more egg clusters; egg cluster, an accumulation of eggs that represents an unspecified number of clutches; clutch, an accumulation of eggs produced by a single female during one egg-laying event.

*Institutional Abbreviations:* MCD, Museu de la Conca Dellà, Isona, Catalonia.

*Repository numbers:* MCD4885, MCD5023-5030, provisionally housed at Museu de la Conca Dellà, Isona, Catalonia.

## Results

### Eggshell Microstructure

The eggshells ranged from 2.23 to 2.91 mm in thickness, with a mean range of 2.40–2.67 mm. Radial thin sections and SEM images of the eggshell revealed a single structural layer of calcite. Radiating spherulites extended from nucleation sites at the inner shell surface until truncated by crystal growth from adjacent nuclei, forming the slightly flared, narrow shell units. The inner shell surface exhibited a pitted appearance, possibly the result of calcium resorption by the embryo or, alternatively, diagenetic dissolution of the eggshell calcite. The spherulitic shell units terminated in rounded nodes (compactituberculate ornamentation *sensu* Mikhaïlov [54]) at the outer shell surface. These nodes were 0.31 mm high and varied from 0.64 to 0.87 mm in diameter. Average node density on the outer shell surface was 239 nodes per cm^2^, and the shell unit height was about 2.8 to 3.3 times its width. The eggshell surface displayed abundant, elliptical pore openings that varied from 65–120 µm in width. These pores were located between the nodes, with a density of approximately 507 pores per cm^2^.

### Site Taphonomy

The fine-grained rocks within the study area dipped steeply to the north at 30°. The egg-bearing strata were discontinuous and thinned to the east, but were laterally traceable for approximately one kilometre [52], [53]. At least nine egg horizons occurred within the measured section, at approximately 0.4, 1.2, 1.8, 3.4, 16.5, 19.5, 23.0, 24.2 and 25.5 meters above the base of the outcrop (Fig. 1B; Table 1). Egg clusters at sites 17E02, 17E03, and 17E05 were laterally adjacent to one another and separated about 3.1 and 4.4 m, respectively. Egg clusters at site 17E04 occurred 6.2 m south of sites 17E03 and 17E05.

**Figure 2:**
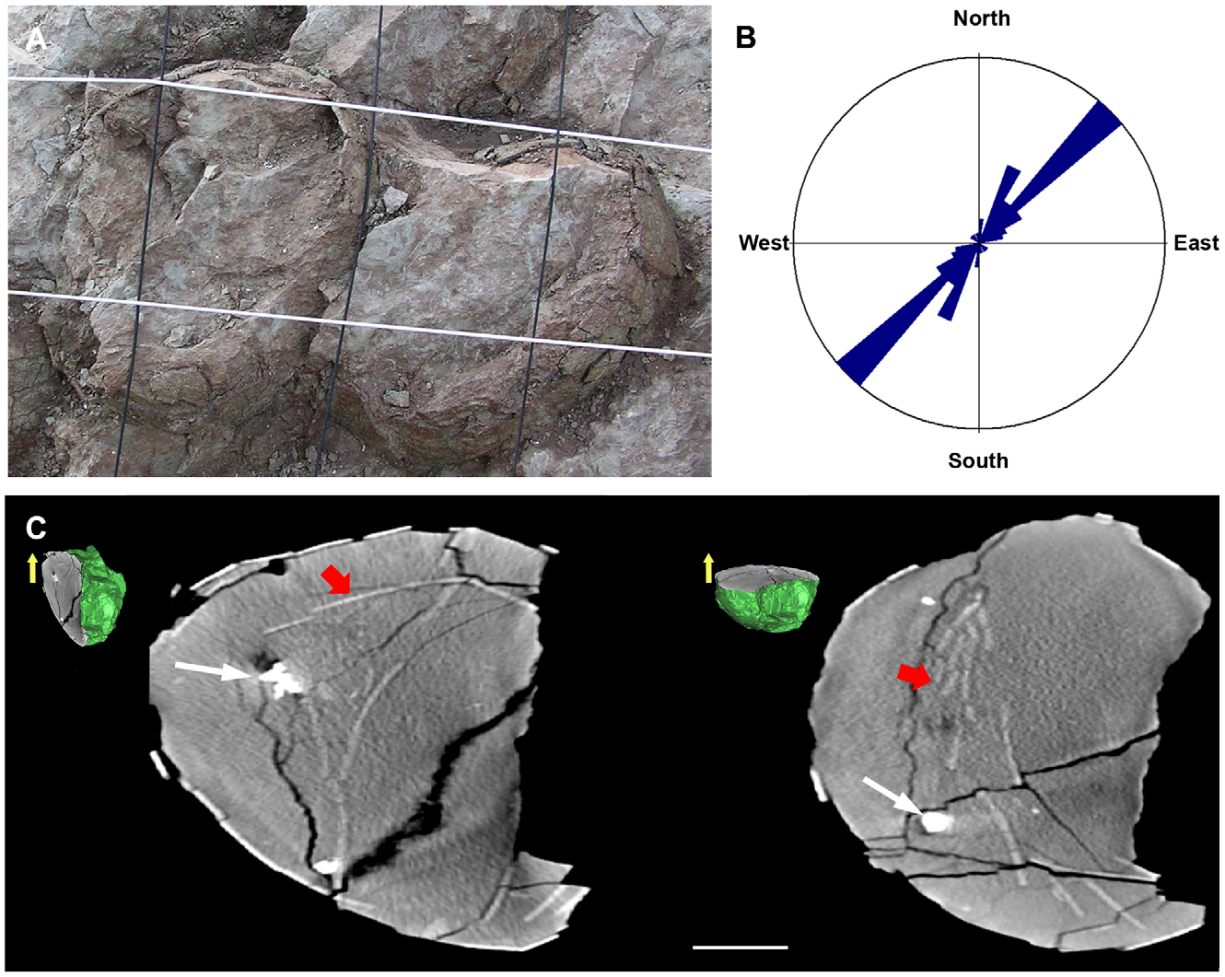
Egg features at Pinyes locality. (A)–Interpreted hatching window in two eggs from clutch 17E04-B. Note the elliptical outline and size of the truncations. Grid squares ∼10 cm. (B)–Rose diagram showing long axis direction for Pinyes eggs. Note the NE-SW alignment, coincident with the tectonic foliation. (C)–Computed Tomography scan images of egg A from cluster 13E02 showing vertical (YZ) cross section (left) and equatorial (XY) cross section (right) with respective three-dimensional miniature render (yellow arrow indicates top of the egg). Note that the grouped eggshell fragments are vertically embedded by sediment. White and red arrows indicate nodules and eggshell pieces, respectively. Scale bars  = 5 cm.

Most eggs exposed at the Pinyes locality were incompletely preserved because of recent erosion; however, excavation occasionally revealed relatively intact specimens in the subsurface. Some eggs exposed in cross-section revealed numerous eggshell fragments, predominantly oriented concave up within the mudstone matrix that filled the egg interior. Two clusters, 17E04-A and 17E04-B contained 8 and 20 eggs, respectively (Table 1). The upper portions of six eggs in 17E04-B appeared truncated and exhibited sizeable areas that lacked eggshell. After correction for dip, this missing eggshell formed an elliptical “opening” parallel to the bedding plane (Fig. 2A).

The mudstones surrounding the eggs displayed extensive bioturbation, minor faults, and penetrative foliation in a NE-SW direction. Eggshell fragments were often displaced and overlap one another, and the eggs exhibited significant deformation due to compression. The general 3-D shape of the eggs was as a scalene ellipsoid in which the relative lengths of the three semi-principal axes were unequal. In the most complete specimen these 3 semi-principal axes measured 10.5 cm, 8.0 cm, and 5.0 cm. Most eggs mapped in the field showed a long axis direction (axis a) of N44°, thus having a general NE-SW orientation which coincided with regional stress fields resulting from tectonic compression (Fig. 2B).

### Computed Tomography

Computed Tomography scans of 14 eggs showed two types of preservation: 1) relatively complete eggs containing no eggshell fragments (eggs C-E from site 13E01, eggs A–E and F from site 18E01, egg E from site 18E02), and 2) eggs with randomly distributed eggshell fragments in the lower third of the egg interior (eggs A–C from site 13E02, egg P from site 17E04). Eggshell fragments in egg P from site 17E04 were oriented concave-up or nearly vertically in the lower third of the egg interior; similarly, eggshell in egg A from site 13E02 were relatively large (up to 7 cm), vertically to sub-vertically oriented, and imbricated. The fragments were tightly grouped, forming a small fragment cluster within the sediment fill. A few ferruginous nodules were also present in some eggs (Fig. 2C).

### Preservation and Clutch Morphology

**Figure 3:**
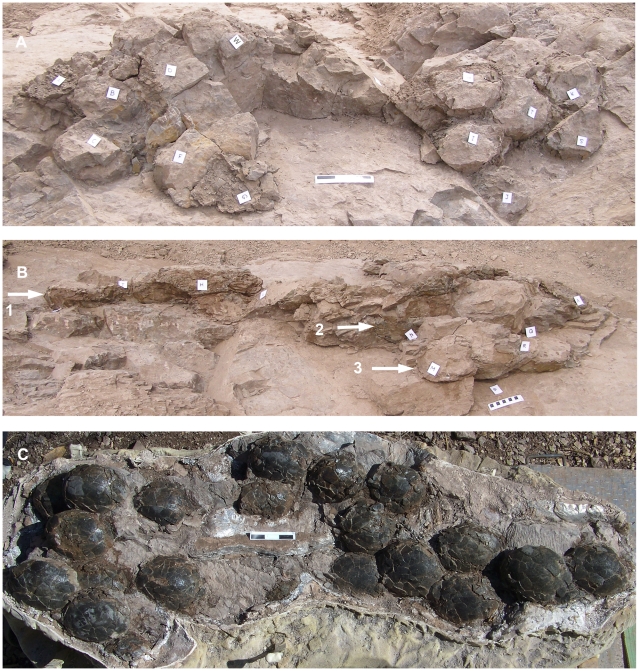
Individual clutches at Pinyes locality. (A)–Field photograph of partially excavated eggs from clutch 18E02, shown from a slightly oblique angle. Scale bar  = 15 cm. (B)–Lateral view of the same clutch. Numbers indicate egg levels. Scale bar  = 10 cm. (C)–Under side of the same clutch as revealed during preparation, shown from an oblique angle. Scale bar  = 15 cm. White labels in A and B indicate eggs.

**Figure 4:**
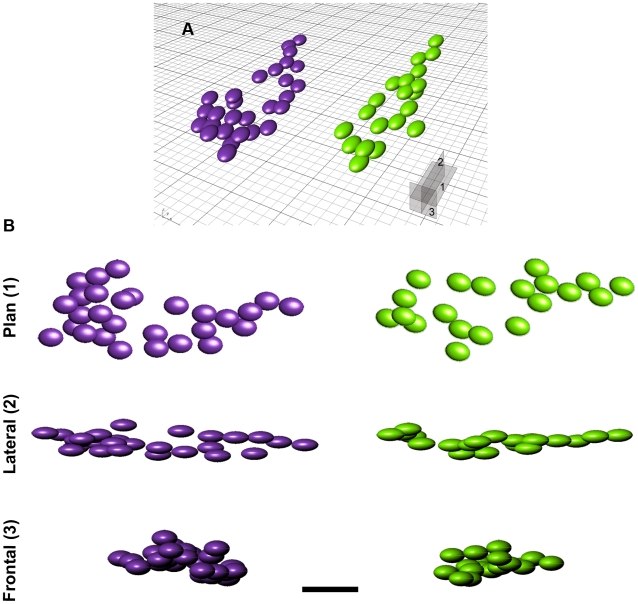
3-D modelling of clutches 18E02 and 17E04-B. (A)–Perspective view of 18E02 (left) and 17E04-B (right), respectively, with indication of the views in B. (B)–Plan, lateral and frontal views for clutches 18E02 and 17E04-B, respectively. Note the similarities between egg arrangements in all views and the bowl-shape, asymmetric geometry. Scale bar  = 50 cm.

Plan-view maps (both 2-D and 3-D representations) of the egg clusters showed one or more of the following eggs arrangements: randomly dispersed, tightly grouped, or linear pattern. Further, the linear and group eggs occurred in the upper and lower portion of the clusters, respectively. These eggs typically occurred in close contact with one another, in two or three superimposed layers (Fig. 3A–C). Moderate to abundant eggshell debris and occasionally large eggshell fragments (>6 cm) were often present in the mudstones that surrounded individual eggs and egg clusters. Trimble Total Station coordinates obtained for eggs in eight clusters allowed detailed reconstruction of the egg locations relative to the bedding plane using Rhinoceros 4.0 ® software (Fig. 4A). Below, we discuss in detail egg clusters 18E02 and 17E04-B, the largest egg accumulations documented at the Pinyes locality (Movie S1). Additional comments about the remaining clusters at sites 17E01, 17E02, 17E03, 17E04-A, 17E05, 18E01, and 18E04 are included as appropriate.

#### Cluster 18E02

This cluster measured 230 cm long, 89 cm wide, and 35 cm deep and consisted of 28 eggs. Plan view (2-D maps and 3-D software reconstructions) showed two patterns of egg arrangement within the cluster: eggs with an elongate distribution and a second area of tightly grouped eggs (Fig. 4B). Direct observation of the prepared cluster in lateral view and 3-D software reconstructions revealed that the eggs occurred in three levels (Fig. 4B). The eggs formed an asymmetric, concave-up, bowl-shaped profile. The superimposed egg levels are far less apparent in traditional 2-D plan-view maps because the excavation intersects the bedding plane and obscures the angle of dip and true clutch geometry. A front view of the cluster in 3-D reconstruction shows similar bowl-shaped geometry with a distinctive asymmetric profile (Fig. 4B).

#### Cluster 17E04-B

This cluster measured 227 cm long, 84 cm wide, and 29 cm deep and contained at least 20 eggs, with 5–6 additional egg likely concealed in the surrounding mudstone. Similar to 18E02, an elongate distribution and a second area of tightly grouped eggs were apparent in plan view from 2-D maps and 3-D software reconstructions (Fig. 4B). Lateral views possible from field observations and 3-D software reconstructions revealed that the eggs in this cluster also occurred at three levels and formed an asymmetric, concave-up, bowl-shaped profile, both in lateral and front views (Fig. 4B).

The remaining clusters at sites 17E02, 17E04-A, 17E05, 18E01, and 18E04 contained 8 to 18 eggs. In plan-view these clusters also showed grouped, linear, or randomly dispersed egg arrangements (Table 1; [53]). In some cases (e.g. cluster at site 18E04), the eggs occurred in one or two superimposed levels.

## Discussion

We assign the Pinyes eggs to *Megaloolithus siruguei* on the basis of egg size, shape, eggshell microstructure, tuberculate ornamentation, and the presence of transversal canals in a tubocanaliculate pore system [36], [53], [55], [56]. The latter represents an unequivocal feature of this oospecies [57].

Eggs and eggshells of *Megaloolithus siruguei* are well documented from various localities in northern Catalonia and southern France [15]–[18], [31], [55], [58]. Egg horizons within the Tremp Formation were once continuous within the basin that extended east to west before the collision of the European and Iberian plates. The uplift of the Pyrenees from Late Cretaceous to Oligocene time produced structural deformation of the egg-bearing strata. The deformation that characterizes the Pyrenees today impacts interpretations of dinosaur reproductive biology. In the following section, we discuss the geologic, biologic, and taphonomic attributes of the nesting locality and their influence on interpretations.

### Geological Attributes

Descriptions of dinosaur nesting localities often provide little information on tectonic deformation in the study areas [1], [3], [8], [13], [15], [16], [19], [23], [26], [27], [29], [59]–[62]. Alternatively, the authors may report the bedding attitude, but behavioural interpretations are made without further reference to correction for dip that results from structural deformation [6], [27], [28], [39].

The dip of the strata in mountainous regions can contribute to misinterpretation of reproductive behaviour. For example, clusters at sites 17E02-17E06 at Pinyes locality occur at the same stratigraphic level; however, site 17E04 appears topographically higher on the outcrop. Disregarding the 30° dip could result in misinterpretation of this single stratum containing the fossil eggs as multiple egg-bearing horizons, which are often interpreted as evidence for “site fidelity”.

The steeply dipping beds at the Pinyes locality also impact other egg and clutch attributes. Spherical egg shape is considered as diagnostic for *Megaloolithus siruguei* [55]; however, egg shape reported from European localities varies from round to sub-round to elliptical (e.g., [16], [18], [19]). The reason for this variation may relate to the orogenic belt in which most of these eggs occur. The angle at which the erosion plane intersects the specimen determines the *apparent* shape and size of the exposed egg. Laboratory preparation and CT imaging of the Pinyes eggs provide a more accurate means of assessment. For example, egg shape approximates a scalene ellipsoid. The long axis direction (axis a) of most eggs follows a general NE-SW orientation in the study area, which coincides with orientation of tectonic foliation in the region (Fig. 2B). This clearly indicates that the shape of the egg was strongly influenced by tectonic processes, as well as the reproductive anatomy of the female. Therefore, it is important to note geologic processes such as deformation in the site description when such processes may adversely impact measurements of egg size, shape, and volume.

### Biological Attributes

Several features documented from the Pinyes locality suggest biological processes also influenced the site taphonomy. For example, some eggs at this locality are intact and relatively complete. Computed tomography scans of some eggs show neither shell fragments nor embryonic skeletal material within the egg interior; therefore, we interpret these specimens as unhatched or infertile eggs. In contrast, the upper surfaces of other eggs exhibit sizeable areas that lack eggshell. These elliptical “openings” in the egg surfaces lie parallel to the bedding plane and correspond to the elliptical shape of the compressed eggs (Fig. 2A). This indicates that both the eggs and openings were modified by tectonic compression. Computed tomography scans also show that these eggs contain multiple eggshell fragments, some of which are very large. These fragments are randomly distributed and concave-up to vertically oriented within the sediment-filled specimens (Fig. 2C). The eggshell fragments entered the egg simultaneously with sediment and as a consequence do not rest directly on the bottom of the egg interior. The similarity of the opening size, shape, and location on the eggs suggest a similar origin. We cautiously interpret this feature as the “hatching window”, as first proposed by Cousin et al., [26].

The hatching window [26] is further supported by CT scans and measurements of similar egg features [63]. Furthermore, Mueller-Töwe et al. [63] suggested that titanosaurs, like many modern egg-laying amniotes, may have possessed an “egg tooth”. Presumably, the embryo used this egg tooth to perforate the shell, thereby producing a large opening in the upper egg surface. They also suggested that large fragments of eggshell preserved within the sediments that filled the eggs indicated that the hatchlings likely escaped the egg while it was covered by sediment, providing strong evidence for underground incubation and hatching.

These explanations for the opening in the upper egg surfaces and eggshell fragments preserved within the egg interior seem plausible. However, this interpretation requires caution for several reasons. First, actualistic experiments [64] reveal that a large opening in the upper egg surface may also result from gas collection and expansion, due to the decay of organic matter in buried eggs. Similarly, some infertile bird and crocodilian eggs developed holes when buried that were similar to the hatching windows reported in fossil eggs [65]. Finally, although taphonomic studies of modern avian sites are well documented [66], [67], crocodilians or turtles nesting sites have not been documented or compared to fossil egg localities.

### Preservation and Clutch Morphology

Three types of egg clusters are preserved at the Pinyes locality (Table 1). Type 1 consists of 20–28 eggs that occur in close association or touching one another. These eggs form a linear and grouped pattern in the upper and lower portion of the cluster, respectively. Type 2 consists of moderate to large clusters of 8 to 18 eggs; the geometry and close egg contact in these egg accumulations are similar to portions of Type 1 clusters. Finally, Type 3 clusters include small accumulations of up to 5 eggs. These specimens, however, were not fully excavated and therefore provide inadequate data for assessment of clutch size and morphology.

The clusters at sites 18E02 and 17E04-B represent Type I preservation; both are interpreted as *in situ* egg clutches, each produced by a single individual. A previous interpretation suggested that eggs in 17E04-B represented multiple, superimposed clutches [52]. However, interpretations of 17E04-B and 18E02 changed with additional preparation and inclusion of 200 additional total station data points for 18E02. Our interpretation is further supported by the similar bowl-shaped geometry, close egg contact, and three egg levels that characterize both clutches. Further, both clutches display a consistent pattern of egg distribution that includes both linear and grouped eggs. Cross sectional, frontal, and plan views are also similar in morphology and dimensions (Fig. 4 and Movie S1). Type 2 clusters at the Pinyes locality, although smaller in size, also show strong similarities to portions of the larger Type 1 clutches. These similarities include multiple egg levels, close egg contact, and more importantly, the combined linear and grouped egg pattern within the clusters. We interpret these smaller (<18 eggs) Type 2 clusters as remnants of larger, eroded clutches.

### Comparisons to Previously Described Clutches

**Figure 5:**
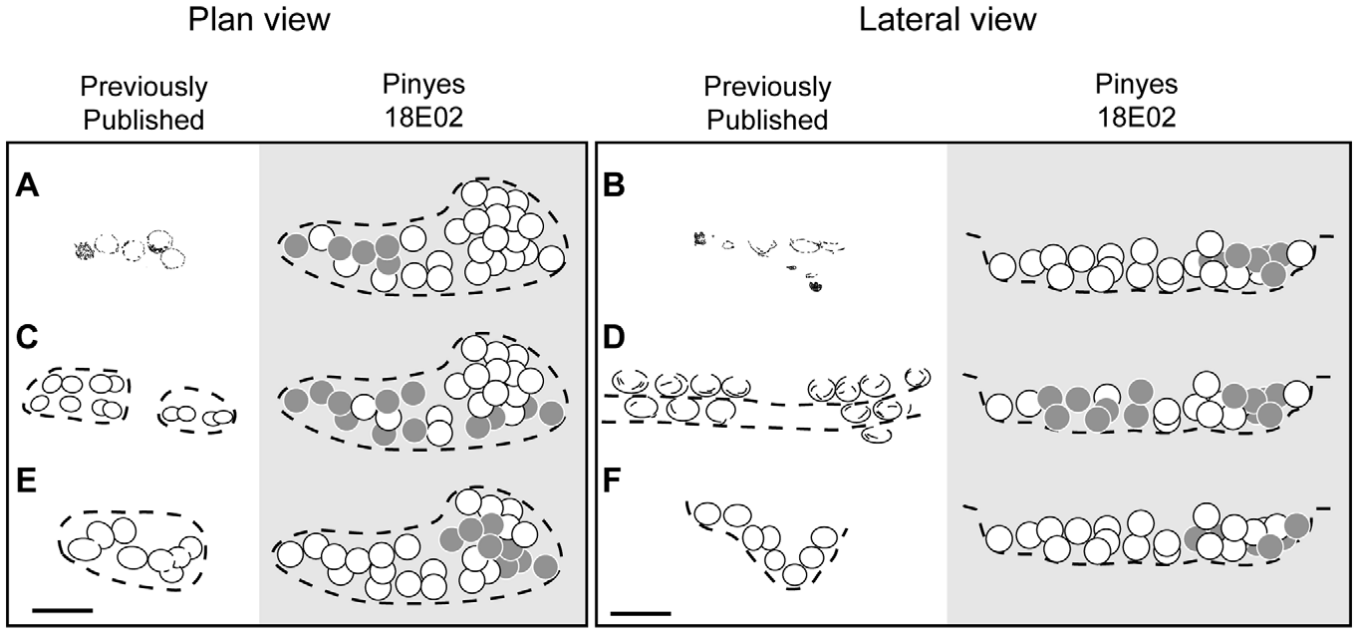
Comparisons of egg arrangements at Pinyes with clutch morphology reported from Europe. (A, C, and E)–Plan view arrangements from Sander et al., [6], Grigorescu et al., [8] and Kérourio [25], and corresponding interpretation of egg arrangements after the Pinyes new clutch morphology, respectively. (B, D, and F)–Lateral view arrangements from the same authors, and interpreted egg arrangements after the Pinyes new clutch morphology, respectively. Scale bar  = 50 cm.

Moratalla and Powell [68] summarized megaloolithid nesting strategies reported in the literature and noted three patterns of egg arrangement: 1) circular pattern of 6–8 eggs with random distribution and conical shape, 2) a linear pattern, and 3) eggs arranged in arcs, which if connected would form circles containing fifteen to twenty eggs. However, the arc pattern [69], [70] has been questioned by some workers [6], [7]. The morphology of clutches at Pinyes sites 18E02 and 17E04-B share both similarities and differences with previous descriptions. For example, the linear arrangement of eggs documented at several localities by Sander and colleagues [6], Grigorescu and colleagues [8], and Kérourio [25] (also see Table 2) corresponds to the upper level of 18E02 and 17E04-B (Fig. 5A, C, D). The same authors report grouped arrangements that are comprised of 6–10 eggs that form an inverted cone-shaped arrangement in cross section and include 2–3 superimposed egg levels. This pattern of egg distribution likely corresponds to the lower, deeper level of the Pinyes clutches (Fig. 5B, D–F).

**Table 2:** Principal localities with megaloolithid clutches indicating number of eggs per “clutch”, egg levels and egg arrangement.

Locality	Clutch size	Egg Levels	Egg Arrangement	References
Aix en Provence (EUR)	25	-	-	[56]
Albas (EUR)	15	2	Linear + grouped	[20], [40]
Basturs-1 (EUR)	7	-	Linear, grouped	[27]
Biscarri (EUR)	7	2	Linear	[15]
Bouches-du-Rhône area (EUR)	15–20	-	Linear	[71]
Clos-la-Neuve (EUR)	7	-	-	[72]
“Coll de Nargó” (EUR)	6	various	Linear, grouped	[6], [28]
Faidella (EUR)	≤15	-	-	[16]
Font del Bullidor (EUR)	16	3	Linear, grouped	[31]
Founbit-Rennes-Le-Château (EUR)	8	1	Arc	[40]
Grande Marquise (EUR)	5	1	Arc	[56]
La Cairanne (EUR)	<8	-	-	[56]
Lavaldieu (EUR)	7	various	-	[56]
Les Bréguières (EUR)	4	1	Grouped	[56]
Mèze (EUR)	6	various	-	[56]
Rousset-sur-Arc (EUR)	8	3–4	Linear + grouped	[25]
St André de Roquelongue (EUR)	8	1	Grouped	[56]
St Laurent (EUR)	6	2	Grouped	[56]
Sextius-Mirabeau (EUR)	<4	1	Linear	[20]
Suterranya-1 (EUR)	6	1	Grouped	[60]
Tustea Densus (EUR)	14	3	Linear + grouped	[8]
Auca Mahuevo (SAM)	∼25–35	2	-	[36]
Salitral Ojo de Agua (SAM)	8, 12, ≤18	1–2	Linear + grouped	[1], [2]
Berthe IV, Salitral de Santa Rosa (SAM)	14	-	Grouped	[21]
Bara Simla Hill, Pat Baba Mandir (IND)	2–7	-	Linear, grouped	[3]
Balasinor Quarry, Jetholi, Dhuvadiya (IND)	3–12	1	Linear, grouped	[3], [62]
Jabalpur (IND)	10–12	-	-	[73]
Khempur (IND)	13	1–2	Grouped	[74]
Pavna (IND)	≤18	1	Linear, grouped	[14]
Rahioli (IND)	10	1	Linear, grouped	[59], [62]

EUR: Europe, SAM: South-America, IND: India.

**Figure 6:**
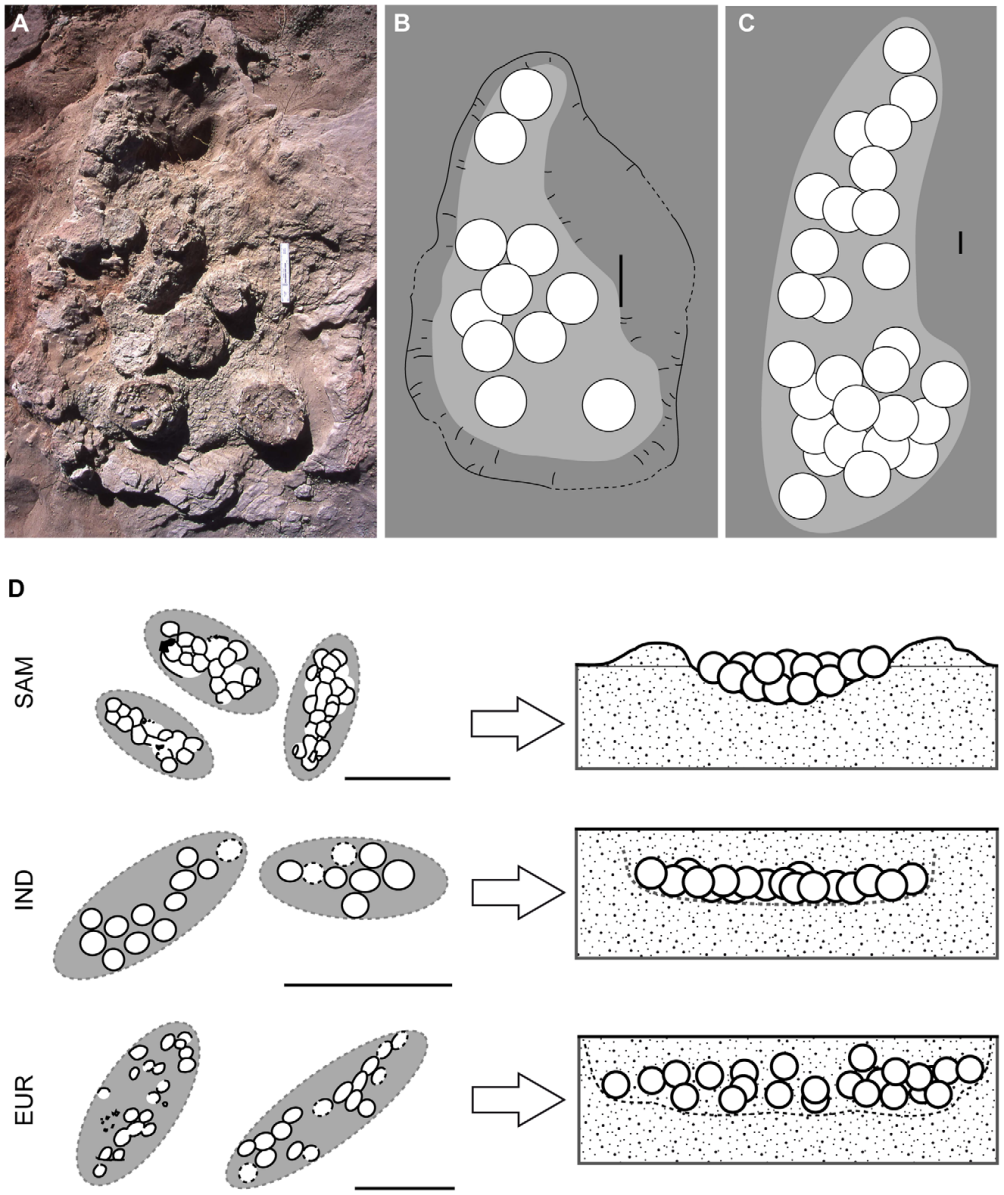
General pattern in megaloolithid clutches. (A)–Field photograph of partially excavated titanosaur nest (NE-01) from Auca Mahuevo locality, Argentina. Scale bar  = 15 cm. (B)–Scheme for the same nest (C)–Pinyes clutch (18E02) morphology inferred after the 3-D model. Note the strong similarity in the elongated kidney-like shape in all three figures. (D)–Published field map and interpreted nest structure in South America (SAM). Modified from Chiappe et al. [37], [75]. (E)–Same for megaloolithid eggs from India (IND). Modified from Mohabey [3], [29]. (F)–Same for megaloolithid eggs from Europe (EUR). Scale bar  = 15 cm (A–C) and 1 meter (D–F).

The pattern of egg distribution in clutches at Pinyes sites 18E02 and 17E04-B resembles that of titanosaurs clutches from the Auca Mahuevo locality in Argentina that contain a similar number of eggs. However, the maximum length for nesting traces at the Auca Mahuevo site is 100–140 cm, which includes the surrounding rim [37], [75], whereas the length for Pinyes clutches (based on the eggs alone) is approximately 230 cm. Nevertheless, the general shape of Pinyes clutches is remarkably similar to the elongate or kidney-shaped structures documented at the Argentine locality (Fig. 6A–C). The general, elongated egg arrangement at the Pinyes site is also similar to several clutches reported in South America and India (Table 2) (Fig. 6D–F).

Clutches 18E02 and 17E04-B are clearly larger than the 5–15 eggs reported by some authors from European, Indian, and South-American localities; however, other workers document clutches containing 15 to 25 eggs per clutch (Table 2), indicating that large clutches are not unusual. It is worth noting that small clutches (<8) reported in the literature typically were not fully excavated (e.g., [6], [16], [27], [28], [60]), which likely prevented full assessment of the clutch size. In addition, several authors report multiple egg levels similar to that documented in the Pinyes clutches (i.e., [2], [6], [8], [15], [25], [37], [40], [56]). Sander and colleagues [6] noted the high rates of erosion in the badlands near Coll de Nargó, and suggested that clutches of less than three eggs represented eroded remnants of once larger clutches. We concur and further suggest that smaller clutches reported at other localities (e.g. Biscarri, “Coll de Nargó”, Faidella, Saint André de Roquelongue, Salitral Ojo de Agua, Bara Simla Hill and Pat Baba Mandir, among others; see Table 2) may also reflect partial preservation of larger clutches that were truncated by recent erosion. Although available data are limited, we suggest that 25 eggs may represent a typical size for megaloolithid clutches, based on the two large clutches at the Pinyes locality and those listed in Table 2.

### Mode of Incubation and Nesting Behaviour

Interpretations regarding nest construction and incubation strategy employed by extinct taxa typically rely on three lines of evidence: 1) nesting traces, 2) superimposed eggs within a clutch, and 3) water vapour conductance (G_H2O_) calculated from fossil eggs. Trace fossil nests are rare in the fossil record [37], [76], and most megaloolithid localities provide little lithologic evidence of nest architecture. To date, interpretations of a “nesting hole” related to megaloolithid egg incubation have been based on observations of superimposed eggs mapped in plan view, cross sectional maps, or from high-resolution 3-D models [31], [40], [41]. With a few exceptions [15], [22], [23], nearly all previous studies infer substrate burial of megaloolithid eggs [3], [6], [8], [13], [20], [25], [27], [28], [29], [31]–[33], [60]. In addition, nearly all studies that calculated G_H2O_ on megaloolithid eggs support substrate burial [8], [32], [34]–[36]. The Auca Mahuevo eggs, however, exhibit significantly lower G_H2O_ than other megaloolithid eggs, indicating they were not buried in a substrate [36].

Evidence from the Pinyes locality corroborates previous studies that conclude that the taxa laying megaloolithid eggs (presumably titanosaurs) were laid in shallow excavated “pit-like”, bowl-shaped, or saucer-like structures. Seymour ([32]; p.9) proposed an upper limit of 13 eggs for the size of a buried sauropod clutch; however, he noted that this number of eggs was small compared to body weight. We suggest that 25 eggs reported at Pinyes and Aix en Provence locality (Table 2) probably represent a typical clutch size for European titanosaurs. Furthermore, there is no compelling evidence that small egg accumulations (<15) represent one of several clutches laid by the same female in a single nesting season (contra [6], [32], [77]). The similarity of shape and 2-D and 3-D geometry of these small egg accumulations to parts of large clutches further supports our interpretation.

### Nest Shape and Pes Morphology

**Figure 7:**
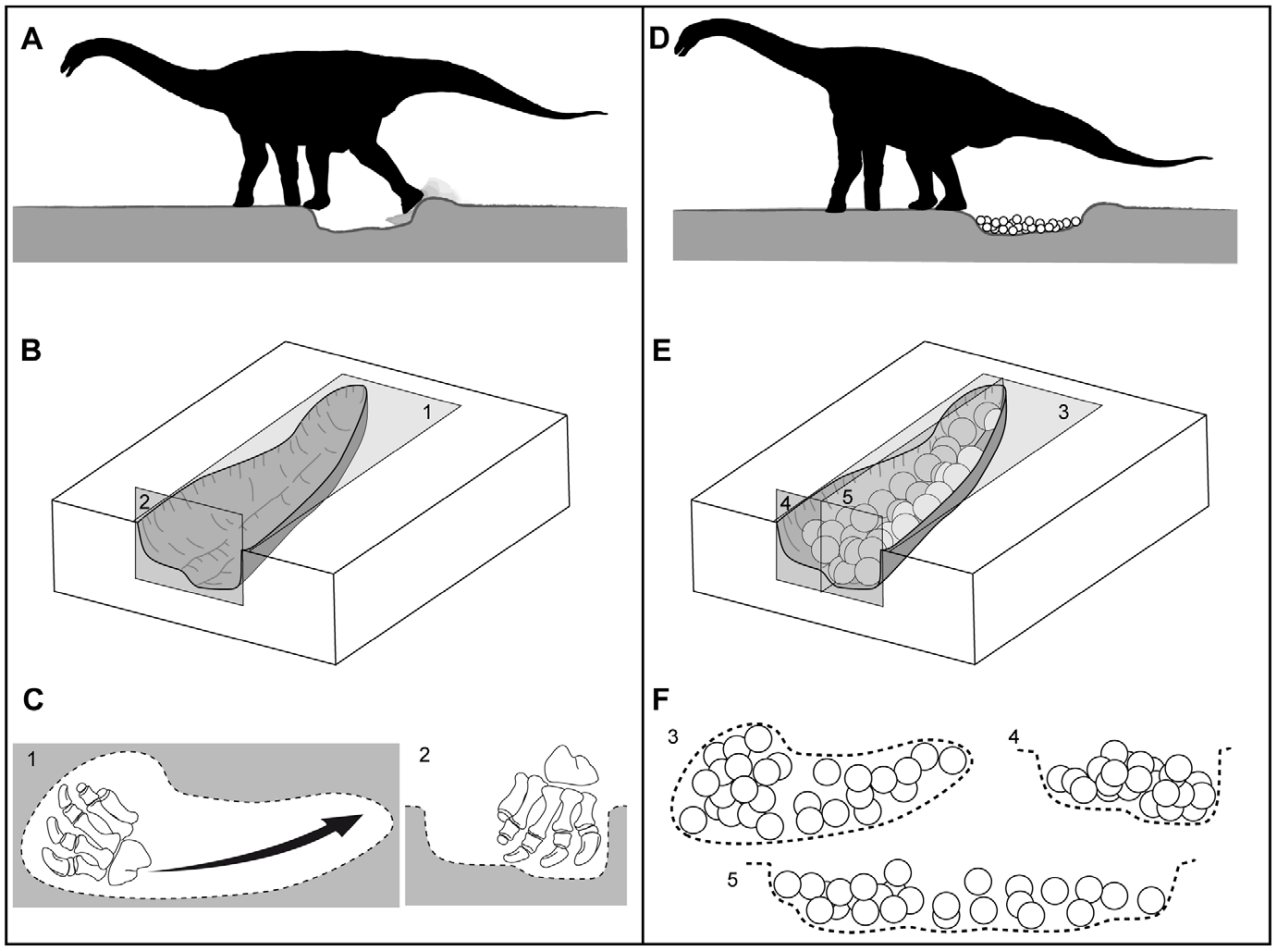
Nest excavation and egg laying. (A)–Titanosaur female using back foot to excavate nest. (B)–Block diagram showing asymmetrical nest morphology in plan (1) and cross section (2) view. (C)–Titanosaur pes and excavated pit in plan and cross section view (1, 2, respectively). Note the asymmetric feature of the excavated hole in frontal and plan views and reconstructions from Figure 4. Pedal reconstruction after [82]. (D)–Egg clutch produced in mass by female. (E)–Block diagram of nest and eggs showing asymmetrical egg distribution in plan (3), frontal (4) and lateral (5) views. (F)–Egg arrangement within the nest from three views.

The remarkably elongated pattern of the Pinyes clutches and their morphologic similarity to clutches of similar size from South America, Europe, and India (Fig. 6D–F) suggest a common nesting behaviour. The flattened pes claws of sauropod dinosaurs appear well suited for movement of sediment during nest excavation [78]. Gallup [78] suggested sauropods may have used scratch-digging for nest excavation. Fowler and Hall [79] concurred with this interpretation, on the basis of their study of the similar ungual morphology and scratch-digging behaviours observed in modern tortoises during nest excavation. This digging behaviour in titanosaur sauropods likely produced the elongated and shallow pits documented at the Pinyes and other localities (Fig. 7A, B).

Articulated specimens (i.e., *Ophistocoelicaudia*, *Epachthosaurus*, and MUCPv-1533 material from La Invernada locality [80]–[82]) allow reconstruction of these titanosaur pedes. Morphological analyses indicate that the articular surfaces of the unguals are inclined, suggesting mobility in vertical and horizontal planes [78], [79], [81]–[83]. We hypothesize that the “kidney shaped” morphology reported in Auca Mahuevo nests and Pinyes clutches resulted from the scratch-digging movement produced by the rear foot of the female. More importantly, the asymmetrical profile of the egg arrangement and thus that of the excavated hole is clearly distinct in frontal and plan views (Fig. 4B, G, and Fig. 7C, E, F), and probably reflects the asymmetrical nature of the pes. Thus, the deeper area of the excavated hole could account for the scratching action produced by the more pronounced inner digits (I - III) of the pes (Fig. 7C).

### Summary and Conclusions

Three dimensional modelling of megaloolithid eggs augmented by traditional 2-D maps and detailed taphonomic study in the Upper Cretaceous Pinyes locality provide more accurate information on clutch geometry and reproductive behaviour. Pinyes clutches exhibit up to 28 eggs in three superimposed levels and the eggs occur in linear and grouped patterns within an elongate, shallow, bowl-shaped depression. We suggest that 25 eggs may represent a typical megaloolithid clutch size. Small egg clusters that display linear or grouped egg arrangements reported at Pinyes and other localities likely reflect recent erosion. The distinct clutch geometry reported at Pinyes and other megaloolithid localities worldwide strongly suggests a common reproductive behaviour that resulted from the use of the hind foot for scratch-digging during nest excavation.

## Supporting Information

Movie S1 3-D modelling of megaloolithid clutches 18E02 and 17E04-B from Pinyes locality.
(0.69 MB MOV)

## References

[1] Powell JE (1992). Hallazgo de huevos asignables a dinosaurios titanosáuridos (Saurischia, Sauropoda) de la Província de Río Negro, Argentina.. Acta Zool Lil.

[2] Powell JE (1996). Nuevos datos sobre huevos de dinosaurios del Cretácico Superior de Río Negro, Argentina.. Ameghiniana.

[3] Sahni A, Tandon S, Jolly A, Bajpai S, Sood A, Carpenter K, Hirsch KF, Horner JR (1994). Upper Cretaceous dinosaur eggs and nesting sites from the Deccan volcano-sedimentary province of peninsular India.. Dinosaur Eggs and Babies.

[4] Manera de Bianco T (1996). Nueva localidad con nidos y huevos de dinosaurios (Titanosauridae) del Cretácico superior, Cerro Blanco, Yaminué, Río Negro, Argentina.. Asociación Paleontológica Argentina, Reunión Argentina de Icnología Publicación Especial.

[5] Horner JR (2000). Dinosaur reproduction and parenting.. Annu Rev Earth Pl Sc.

[6] Sander PM, Peitz C, Jackson FD, Chiappe LM (2008). Upper Cretaceous titanosaur nesting sites and their implications for sauropod dinosaur reproductive biology.. Palaeontogr Abt A.

[7] Carpenter K (1999). Eggs, nests and baby dinosaurs..

[8] Grigorescu D, Weishampel D, Norman D, Seclamen M, Rusu M, Carpenter K, Hirsch KF, Horner JR (1994). Late Maastrichtian dinosaur eggs from the Hateg Basin (Romania).. Dinosaur Eggs and Babies.

[9] Grigorescu D (2006). Hatchlings of *Telmatosaurus transsylvanicus* (Ornithischia, Hadrosauridae) associated with megaloolithid eggs in the Tustea nesting site (Hateg Basin, Romania).. Ameghiniana.

[10] Chiappe LM, Coria RA, Dingus L, Jackson F, Chinsamy A (1998). Sauropod dinosaur embryos from the Late Cretaceous of Patagonia.. Nature.

[11] Chiappe LM, Salgado L, Coria R (2001). Embryonic skulls of titanosaur sauropod dinosaurs.. Science.

[12] Chiappe L, Dingus L, Jackson F, Grellet-Tinner G, Aspinall R, Bravo AM, Reyes T (2000). Sauropod eggs and embryos from the Late Cretaceous of Patagonia.. First International Symposium on Dinosaur Eggs and Babies- Extended Abstracts.

[13] Faccio G, Carpenter K, Hirsch KF, Horner JR (1994). Dinosaurian eggs from the Upper Cretaceous of Uruguay.. editors Dinosaur Eggs and Babies.

[14] Mohabey DM (1996). A new oospecies, *Megaloolithus matleyi*, from the Lameta Formation (Upper Cretaceous) of Chandrapur district, Maharashtra, India, and general remarks on the palaeoenvironment and nesting behavior of dinosaurs.. Cretaceous Research.

[15] López-Martínez N, Moratalla JJ, Sanz JL (2000). Dinosaurs nesting on tidal flats.. Palaeogeogr, Palaeocl.

[16] Bravo AM, Moratalla JJ, Santafé JV, Santisteban C, Bravo AM, Reyes T (2000). Faidella, a new Upper Cretaceous nesting site from the Tremp basin (Lérida province, Spain).. First International Symposium on Dinosaur Eggs and Babies- Extended Abstracts.

[17] Bravo AM, Vila B, Galobart À, Oms O (2005). Restos de huevos de dinosaurio en el sinclinal de Vallcebre (Berguedà, Provincia de Barcelona).. Rev Esp Paleont.

[18] Garcia G, Vianey-Liaud M (2001). Nouvelles données sur les coquilles d'oeufs de dinosaures Megaloolithidae du Sud de la France: systématique et variabilité intraspécifique.. CR Acad Sci II A.

[19] Codrea V, Smith T, Dica P, Folie A, Garcia G (2002). Dinosaur egg nests, mammals and other vertebrates from a new Maastrichtian site of the Hateg basin (Romania).. C R Palevol.

[20] Cousin R (2002). Organisation des pontes de dinosauriens de la parafamille des Megaloolithidae Zhao, 1979.. Bulletin trimestriel de la Société géologique de Normandie et des Amis du Muséum du Havre.

[21] Salgado L, Magalhaes Ribeiro C, García RA, Fernández MS (2009). Late Cretaceous megaloolithid eggs from Salitral de Santa Rosa (Río Negro, Patagonia, Argentina): inferences on the titanosaurian reproductive biology.. Ameghiniana.

[22] Dughi R, Sirugue F (1958). Observations sur les oeufs de dinosaurs du bassin d'Aix-en- Provence: les oeufs à conquilles bistratifiées.. CR Acad Sci.

[23] Cousin R, Breton G, Fournier R, Watte JP (1989). Dinosaur egg-laying and nesting: the case of an upper Maastrichtian site at Rennes-Le-Chateau (Aude, France).. Hist Biol.

[24] Freytet P (1965). Découverte d'oeufs de Dinosaures à Saint-André-de-Roquelongue (Aude).. Bulletin de la Sociéte d'Études de la Science de l'Aude.

[25] Kérourio P (1981). Nouvelles observations sur le mode de nification et de ponte chez les dinosauriens du Crétacé Terminal du Midi de la France.. Comptes Rendus Sommaire des Séances de la Société Géologique de France.

[26] Cousin R, Breton G, Fournier R, Watté JP, Carpenter K, Hirsch KF, Horner J (1994). Dinosaur egglaying and nesting in France.. Dinosaur Eggs and Babies.

[27] Sanz JL, Moratalla JJ, Díaz-Molina M, López-Martínez N (1995). Dinosaur nests at the sea shore.. Nature.

[28] Sander PM, Peitz C, Gallemí J, Cousin R (1998). Dinosaurs nesting on a red beach?. CR Acad Sci II A.

[29] Mohabey DM, Tidwell V, Carpenter K (2005). Late Cretaceous (Maastrichtian) nests, eggs, and dung mass (Coprolites) of sauropods (titanosaurs) from India.. Thunder Lizard: The Sauropodomorph Dinosaurs.

[30] Grellet-Tinner G, Chiappe L, Norell M, Bottjer D (2006). Dinosaur eggs and nesting behaviors.. Palaeogeogr, Palaeocl.

[31] Vila B, Galobart À, Oms O, Poza B, Bravo AM (2009). Assessing the nesting strategies of Late Cretaceous titanosaurs: 3D-clutch geometry from a new megaloolithid eggsite.. Lethaia.

[32] Seymour RS (1979). Dinosaur egg: gas conductance through the Shell, water loss during incubation and clutch size.. Paleobiol.

[33] Erben HK, Hoefs J, Wedepohl KH (1979). Paleobiologic and isotopic studies of eggshells from a declining dinosaur species.. Paleobiol.

[34] Williams DLG, Seymour RS, Kérourio P (1984). Structure of fossil dinosaur eggshell from the Aix Basin, France.. Palaeog, Palaeocl.

[35] Deeming DC (2006). Ultrastructural and functional morphology of eggshells supports the idea that dinosaur eggs were incubated buried in a substrate.. Paleontology.

[36] Jackson F, Varricchio J, Jackson R, Vila B, Chiappe L (2008). Comparison of water-vapor conductance on a titanosaur egg from Argentina with a *Megaloolithus siruguei* from Spain.. Paleobiol.

[37] Chiappe LM, Schmitt JG, Jackson FD, Garrido A, Gingus L (2004). Nest structure for sauropods: sedimentary criteria for recognition of dinosaur nesting traces.. Palaios.

[38] Mohabey DM (1984). Study of dinosaurian eggs from Infra-trappean Limestone in Kheda district, Gujarat.. J Geol Surv India.

[39] Garcia G, Dutour Y, Cojan I, Valentin X, Cheylan G (2003). Long-term fidelity of megaloolithid egg-layers to a large breeding-ground in the upper Cretaceous of Aix-en-Provence (southern France).. Palaeovertebrata.

[40] Cousin R, Breton G, Bravo AM, Reyes T (2000). A fine and complete excavation is necessary to demonstrate a dinosaur clutch structure.. First International Symposium on Dinosaur Eggs and Babies- Extended Abstracts.

[41] Fortuny J, Vila B, Galobart À, Cambra O, Martínez-Pérez C, Chamero B, Escaso F, de Esteban S, Marugán J (2007). Técnicas de documentación y representación tridimensional de puestas de dinosaurio.. Cantera Paleontológica.

[42] Puigdefàbregas C, Souquet P (1986). Tecto-sedimentary cycles and depositional sequences of the Mesozoic and Tertiary from the Pyrenees.. Tectonophysics.

[43] Cámara P, Klimowitz J (1985). Interpretación geodinámica de la vertiente centro-oriental surpirenaica (Cuencas de Jaca-Tremp).. Estudios Geológicos.

[44] Muñoz JA, McKlay KR (1992). Evolution of a Continental Collision Belt: ECORS Pyrennes Crustal Balanced Cross-section.. Thrust Tectonics.

[45] Díaz Molina M (1987). Sedimentación sintectónica asociada a una subida relativa del nivel del mar durante el Cretácico superior (Fm.. Tremp, Provincia de Lérida). Estud Geol, volumen extraordinario Galve-Tremp.

[46] Ardèvol L, Klimowitz J, Malagón J, Nagtegaal PJC (2000). Depositional sequence response to foreland deformation in the Upper Cretaceous of Southern Pyrenees.. Am Assoc Petr Geol B.

[47] Rosell J, Linares R, Llompart C (2001). El “Garumniense” Prepirenaico.. Rev Soc Geol España.

[48] Riera V, Oms O, Gaete R, Galobart À (2009). The end-Cretaceous dinosaur succession in Europe: The Tremp Basin record (Spain).. Palaeog, Palaeocl.

[49] Mey PWH, Nagtegaal PJC, Roberti KJ, Hartevelt JJA (1968). Lithostratigraphic subdivision of Post-Hercynian deposits in the South-Central Pyrenees, Spain.. Leid Geol Med.

[50] Vila B, Gaete R, Galobart À, Oms O, Peralba (2006). Nuevos hallazgos de dinosaurios y otros tetrápodos continentales en los Pirineos sur-centrales y orientales: resultados preliminares.. Actas de las III Jornadas Internacionales sobre Paleontología de Dinosaurios y su Entorno, Salas de los Infantes (Burgos).

[51] Feist M, Colombo F (1983). La limite Crétacé-Tertiaire du point de vue des charophytes.. Géol Méd.

[52] Jackson F (2007). Titanosaur reproductive biology: comparison of the Auca Mahuevo titanosaur nesting locality (Argentina), to the Pinyes *Megaloolithus* nesting locality (Spain).. Unpublished PhD dissertation.

[53] Vila B, Jackson F, Galobart À (2010). First data on dinosaur eggs and clutches from Pinyes locality (Upper Cretaceous, Southern Pyrenees). Ameghiniana.

[54] Mikhaïlov KE (1997). Fossil and recent eggshell in amniotic vertebrates: fine structure, comparative morphology and classification.. Spec Pap Palaeontol.

[55] Vianey-Liaud M, Mallan P, Buscail O, Montgelard G, Carpenter K, Hirsch KF, Horner JR (1994). Review of French dinosaur eggshells: morphology, structure, mineral and organic composition.. Dinosaur Eggs and Babies.

[56] Garcia G (1998). Les coquilles d'oeufs de dinosaures du Crétacé supérieur du Sud de la France: diversité, paléobiologie, bichronologie et paléoenvironnement Unpublished PhD dissertation..

[57] Elez J, López-Martínez N, Bravo AM, Reyes T (2000). Interrelationships between growth of mineral phase and pore system in dinosaur eggshells.. First International Symposium on Dinosaur Eggs and Babies- Extended Abstracts.

[58] Vianey-Liaud M, López-Martínez N (1997). Late Cretaceous dinosaur eggshells from the Tremp Basin, southern Pyrenees, Lleida, Spain.. J Paleont.

[59] Srivastava S, Mohabey DM, Sahni A, Pant SC (1986). Upper Cretaceous dinosaur egg clutches from Kheda District (Gujarat, India): their distribution, shell ultrastructure and palaeoecology.. Palaeontogr Abt A.

[60] López-Martínez N, Ardèvol L, Vicens E, Capdevila J, López-Martínez N (1999). Paleontology and petrography.. First International Symposium on Dinosaur Eggs and Babies: Field Trip Guide, Lleida.

[61] Peitz C (2000). Fortpflanzungsbiologische und systematische implikationen von Dinosauriergelegen aus dem Maastricht von Katalonien (NE-Spanien) sowie die Sedimentologie ihrer Fundstellen.. Unpublished PhD dissertation.

[62] Mohabey DM, Bravo AM, Reyes T (2000). Indian Upper Cretaceous (Maastrichtian) dinosaur eggs: their parataxonomy and implication in understanding the nesting behavior.. First International Symposium on Dinosaur Eggs and Babies- Extended Abstracts.

[63] Mueller-Töwe IJ, Sander PM, Schüller H, Thies D (2002). Hatching and infilling of dinosaur eggs as revealed by computed tomography.. Palaeontogr Abt A.

[64] Bravo AM, Buscalioni AD, Merino L, Müller BG (2003). Experimental taphonomy of avian eggs and eggshells: effects on the early diagenesis.. Palaeovertebrata.

[65] Soja C (2008). Unscrambling dinosaur eggs.. American Paleontologist.

[66] Hayward JL, Amlaner CJ, Young KA (1989). Turning eggs to fossil: experiments in taphonomy.. J of Vert Paleont.

[67] Hayward JL, Dickson KM, Gamble SR, Owen AW, Owen KC Eggshell taphonomy: environmental effects on fragment orientation.. Historical Biology.

[68] Moratalla JJ, Powell JE, Carpenter K, Hirsch KF, Horner JR (1994). Dinosaur nesting patterns.. Dinosaur Eggs and Babies.

[69] Beetschen JC (1985). Sur les niveaux à coquilles d'oeufs de dinosauriens de la région de Rennes-le-Château (Aude).. Actes du Colloque Les Dinosaures de la Chine à la France.

[70] Breton G, Fournier R, Watte JP (1986). Le lieu de ponte de dinosaures de Rennes-le-Château (Aude): premiers résultats de la champagne de fouilles 1984.. Annuales du Muséum du Havre.

[71] Dughi R, Sirugue F (1966). Sur la fossilization des oeufs de dinosaurs.. Comptes rendus des séances de l'Académie des Sciences.

[72] Vianey-Liaud M, Garcia G, Bravo AM, Reyes T (2000). The interest of French Late Cretaceous dinosaur eggs and eggshells.. First International Symposium on Dinosaur Eggs and Babies-Extended Abstracts.

[73] Sahni A (2003). Indian dinosaurs revisited.. Current Science.

[74] Mohabey DM, Sahni A (1990). Dinosaur eggs from Lameta Formation of western and central India: their occurrence and nesting behaviour.. Cretaceous Event Stratigraphy and the Correlation of the Indian Non-marine Strata, Contributions from the Seminar cum Workshop I.G.C.P. 216 and 245.

[75] Chiappe LM, Jackson F, Coria RA, Dingus L, Curry-Rogers KA, Wilson JA (2005). Nesting titanosaurs from Auca Mahuevo and adjacent sites: understanding sauropod reproductive behavior and embryonic development.. The Sauropods: Evolution and Paleobiology.

[76] Varricchio DJ, Jackson F, Trueman CN (1999). A nesting trace with eggs for the Cretaceous theropod dinosaur *Troodon formosus*.. J of Vert Paleont.

[77] Seymour RS, Ackerman RA (1980). Adaptations to underground nesting in birds and reptiles.. Am Zool.

[78] Gallup MR (1989). Functional morphology of the hindfoot of the Texas sauropod *Pleurocoelus* sp. indet.. Geological Society of America, Special Paper.

[79] Fowler D, Hall L. Scratch-digging sauropods, revisited: similarity of sauropod pes unguals to tortoises, and inferred nest-excavating behavior.. Museum of the Rockies Occasional Paper.

[80] Borsuk-Bialyncka M (1977). A new camarasaurid sauropod *Opisthocoelicaudia skarzynskii*, gen. n. sp. n. from the Upper Cretaceous of Mongolia.. Palaeont Pol.

[81] Martínez RD, Jiménez O, Rodríguez J, Luna M, Lamanna MC (2004). An articulated specimen of the basal titanosaurian (Dinosauria: Sauropoda) *Epachthosaurus sciuttoi* from the Early Late Cretaceous Bajo Barreal Formation of Chubut Province, Argentina.. J of Vert Paleont.

[82] González-Riga BJ, Calvo JO, Porfiri J (2008). An articulated titanosaur from Patagonia (Argentina): new evidence of neosauropod pedal evolution.. Palaeoworld.

[83] Bonnan MF, Carpenter K, Tidwell V (2005). Pes anatomy in sauropod dinosaurs: implications for functional morphology, evolution, and phylogeny.. Thunder-Lizards: The Sauropodomorph Dinosaurs.

